# The missing link: *ARID1B* non-truncating variants causing Coffin-Siris syndrome due to protein aggregation

**DOI:** 10.1007/s00439-024-02688-9

**Published:** 2024-07-19

**Authors:** Elisabeth Bosch, Esther Güse, Philipp Kirchner, Andreas Winterpacht, Mona Walther, Marielle Alders, Jennifer Kerkhof, Arif B. Ekici, Heinrich Sticht, Bekim Sadikovic, André Reis, Georgia Vasileiou

**Affiliations:** 1 Institute of Human Genetics, Universitätsklinikum Erlangen, Friedrich-Alexander-Universität Erlangen-Nürnberg, 91054 Erlangen, Germany; 2 Amsterdam University Medical Center, University of Amsterdam, Department of Human Genetics, Amsterdam Reproduction and Development Research Institute, Amsterdam, The Netherlands; 3 Verspeeten Clinical Genome Centre, London Health Sciences Centre, London, ON Canada; 4 Division of Bioinformatics, Institute of Biochemistry, Friedrich-Alexander-Universität Erlangen-Nürnberg, 91054 Erlangen, Germany; 5 Department of Pathology and Laboratory Medicine, Western University, London, ON Canada; 6 Centre for Rare Diseases Erlangen (ZSEER), Universitätsklinikum Erlangen, Erlangen, Germany

## Abstract

*ARID1B* is the most frequently mutated gene in Coffin-Siris syndrome (CSS). To date, the vast majority of causative variants reported in *ARID1B* are truncating, leading to nonsense-mediated mRNA decay. In the absence of experimental data, only few *ARID1B* amino acid substitutions have been classified as pathogenic, mainly based on clinical data and their *de novo* occurrence, while most others are currently interpreted as variants of unknown significance. The present study substantiates the pathogenesis of *ARID1B* non-truncating/NMD-escaping variants located in the SMARCA4-interacting EHD2 and DNA-binding ARID domains. Overexpression assays in cell lines revealed that the majority of EHD2 variants lead to protein misfolding and formation of cytoplasmic aggresomes surrounded by vimentin cage-like structures and co-localizing with the microtubule organisation center. ARID domain variants exhibited not only aggresomes, but also nuclear aggregates, demonstrating robust pathological effects. Protein levels were not compromised, as shown by quantitative western blot analysis. *In silico* structural analysis predicted the exposure of amylogenic segments in both domains due to the nearby variants, likely causing this aggregation. Genome-wide transcriptome and methylation analysis in affected individuals revealed expression and methylome patterns consistent with those of the pathogenic haploinsufficiency *ARID1B* alterations in CSS cases. These results further support pathogenicity and indicate two approaches for disambiguation of such variants in everyday practice. The few affected individuals harbouring EHD2 non-truncating variants described to date exhibit mild CSS clinical traits. In summary, this study paves the way for the re-evaluation of previously unclear *ARID1B* non-truncating variants and opens a new era in CSS genetic diagnosis.

**Supplementary Information:**

The online version contains supplementary material available at 10.1007/s00439-024-02688-9.

## Introduction

The BAF (BRG1/BRM-associated factor) complex, also referred to as SWI/SNF complex, is a highly conserved multi-subunit ATP-dependent chromatin remodelling complex, which regulates gene expression by repositioning nucleosomes and mediating DNA accessibility to transcription factors. Its subunits are assembled into different BAF complexes, depending on tissue context and developmental timepoint (Mashtalir et al. 2018; Wanior et al. 2021). Pathogenic variants in genes encoding BAF subunits have been associated with neurodevelopmental delay disorders (NDDs) collectively referred to as BAFopathies. It has recently been shown that BAF complex subunits exhibit the highest average number of *de novo* single nucleotide variants (SNVs) in NDD and autism spectrum disorder (ASD) cohorts, highlighting an important role in neurodevelopment (Valencia et al. 2023).

The most well defined BAFopathy, caused by pathogenic variants in several BAF subunit genes including *ARID1A*, *ARID1B*, *SMARCA4*, *SMARCC2*, *SMARCB1*, *SMARCE1*, *DPF2*, and *BICRA*, is the autosomal dominant Coffin-Siris syndrome (CSS; MIM 135,900) (Hoyer et al. 2012; Santen et al. 2013; Kosho et al. 2014b; Vasileiou et al. 2018; Barish et al. 2020; Vasko et al. 2021; Bosch et al. 2023). The clinical spectrum is highly variable, ranging from mild to severe, partly depending on the affected BAF subunit. Nevertheless, even individuals with variants in the same gene exhibit phenotypic and clinical differences, some even presenting without the characteristic CSS hallmarks (Kosho et al. 2014a, b; van der Sluijs et al. 2019). The most frequently mutated BAF subunit is ARID1B, accounting for 50–83% of CSS cases (Santen et al. 2013; Wieczorek et al. 2013; Tsurusaki et al. 2014; Kosho et al. 2014a), and 1% of all NDD cases (Hoyer et al. 2012; van der Sluijs et al. 2019; Gillentine et al. 2022; Valencia et al. 2023).

ARID1B (AT-rich interactive domain-containing protein 1B) and its paralog ARID1A play a significant role in the stabilization of the BAF complex base module structure (He et al. 2020). The protein contains an AT-rich interacting domain (ARID), as well as two Eld/Osa homology domains (EHD1 and EHD2). The ARID domain is a DNA-binding domain, which indistinctly recognises target sequences, regardless of their specific properties (Wilsker 2004). The C-terminal EHD1 and EHD2 domains are able to interact with each other, presumably leading to the formation of homodimers or ARID1A/B heterodimers. The EHD2 domain also mediates the interaction with SMARCA4 (BRG1), the core subunit of the BAF complex with ATPase activity (Hurlstone et al. 2002; Inoue et al. 2002) (File S1 “domains”).

To date, the vast majority of known pathogenic variants in *ARID1B* are either truncating (nonsense, frameshifting, splice), leading to nonsense-mediated mRNA decay (NMD), or CNVs (deletions or duplications) encompassing exons or the whole gene. Only few individual clinical reports of (likely) pathogenic *ARID1B* missense variants in cases with either a Coffin-Siris/Coffin-Siris-like phenotype or corpus callosum anomalies have been reported (Mignot et al. 2016; Yan et al. 2019; Chevarin et al. 2020; Miyamoto et al. 2021). Their classification was based on clinical assessment, *in silico* predictions and/or *de novo* occurrence. So far, a total of two pathogenic and 16 likely pathogenic missense variants dispersed throughout the whole gene have been listed in the ClinVar database (File S1 “missense_clinvar”), although clinical description or functional evidence is lacking. Overall, the scientific community and clinicians appear cautious concerning the pathogenicity of non-truncating *ARID1B* variants, suggesting that they are rarely causative (Aref-Eshghi et al. 2018a; van der Sluijs et al. 2019). Only recently, by performing large-scale computational and mutational screening assays, Mermet-Meillon and colleagues demonstrated that missense variants in the EHD2 domain of ARID1B lead to protein destabilisation or misfolding (Mermet-Meillon et al. 2024).

Using an alternative approach, we confirmed the pathogenicity of non-truncating or NMD-escaping *ARID1B* variants located in the EHD2 domain. Transcriptome and methylation analysis revealed a transcription profile similar to that of *ARID1B* haploinsufficiency variants and a BAFopathy episignature, respectively. Overexpression of the majority of EHD2 variants in cell lines led to the formation of cytoplasmic aggregates further characterised as aggresomes. Expanding the analysis to non-truncating variants located in the ARID domain showed similar and even more pronounced functional consequences. These findings suggest protein misfolding and stable aggregation as the cause of pathogenicity and support a loss-of-function pathomechanism.

## Materials and methods

### Individuals

**Fig. 1:**
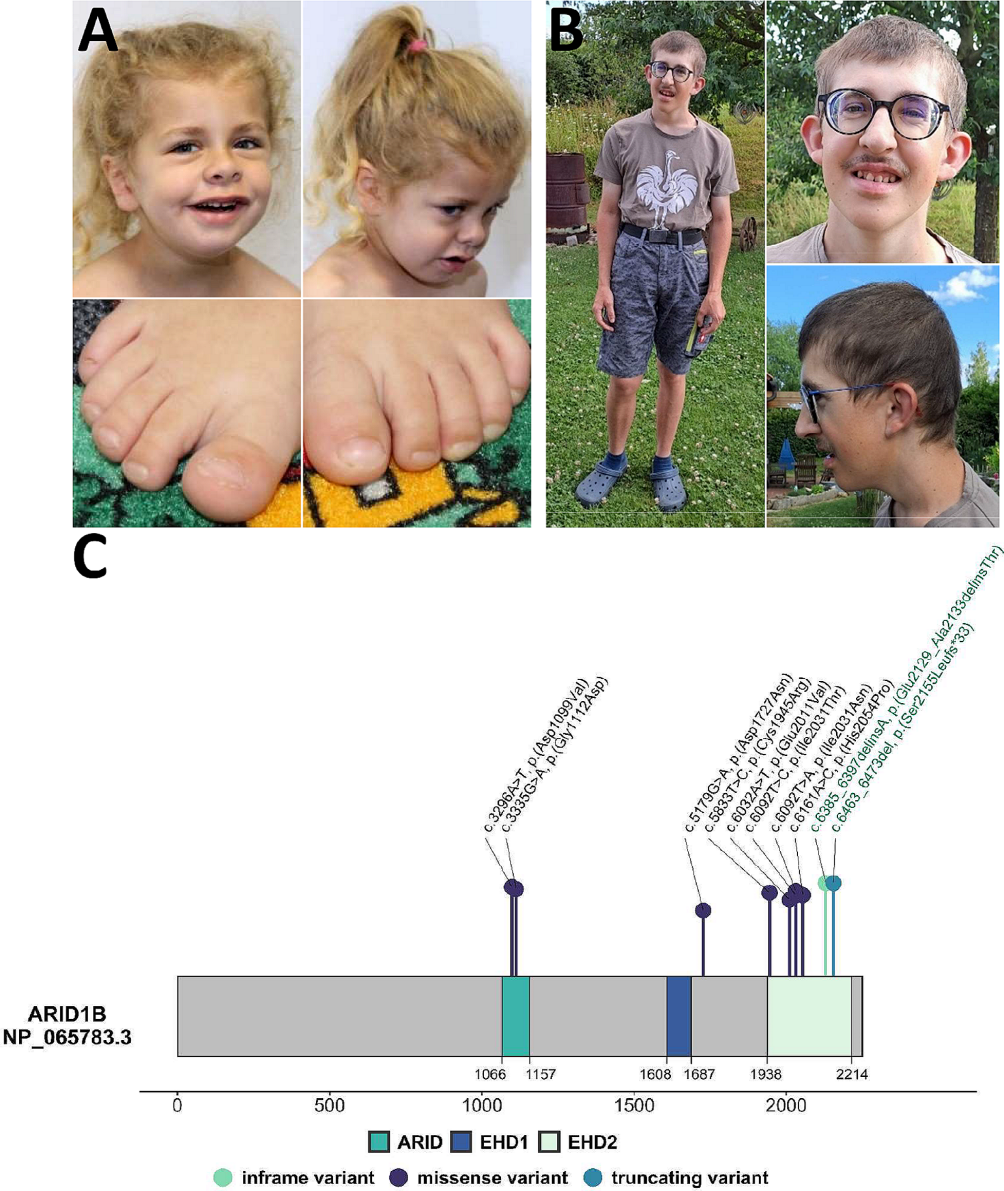
Appearance of *ARID1B* individuals and ARID1B linear protein structure. **(A)** Facial and images from feet of Ind1 (4y7m), carrying the indel variant 2129del4. **(B)** Facial and body images of Ind2 (19y9m), carrying the 2188ter frameshift variant. Note the coarseness of facial features in both individuals. **(C)** Linear model of the ARID1B protein (NP_065783.3) and its domains: a DNA-binding AT-rich interacting domain (ARID, amino acids (aa) 1066–1157), and two Eld/Osa homology domains (EHD1, aa 1608–1687, and EHD2, aa 1938–2214). The EHD2 domain has been shown to mediate interaction with SMARCA4. Circles above the protein model indicate the herein investigated heterozygous non-truncating variants (light green: inframe indel; dark blue: missense; blue: frameshift). For missense variants, their scaled CADD (Combined Annotation Dependent Depletion) (Rentzsch et al. 2019) scores correspond to the lollipop segment length. Variants labelled in green were identified in patients reported herein

The newly reported female individual Ind1-2129del4 and the previously reported male individual Ind2-2188ter (Hoyer et al. 2012) (Fig. 1A-B) were referred to the Human Genetics Institute of the University Hospital Erlangen in Germany for detailed clinical and genetic assessment. Informed written consent for the publication of clinical data and photos were obtained from the legal guardians, and the study was approved by the ethical committee of the medical faculty of the Friedrich-Alexander-Universität Erlangen-Nürnberg.

### Clinical information

The clinical manifestations of Ind1-2129del4 were systematically assessed and together with the clinical features of the previously reported Ind2-2188ter (Hoyer et al. 2012) were standardized according to the HPO terminology. For the latter, novel clinical data were added after re-evaluation at the age of 19 years and 9 months (Fig. 1A-B, File S1 “clinical_table”, File S2 “clinical reports”). A clinical description of previously reported individuals, although incomplete, was available only for Ind5812-H2054P (Miyamoto et al. 2021), Ind11-I2031T (Mignot et al. 2016) and Ind10-I2031N (Yan et al. 2019) and was extracted from the respective publications (File S1 “clinical_table”). Facial dysmorphic features of Ind1-2129del4 and Ind2-2188ter were determined independently by two clinical geneticists and for the previously described cases, when available, from the respective clinical report or analysis of the published pictures (File S1 “clinical_table”, File S2 “clinical reports”).

### Genetic analysis

Following clinical suspicion of CSS, the *ARID1B* insertion-deletion (indel) 2129del4 in Ind1 was identified by Sanger sequencing. Total RNA was extracted from untransformed blood lymphocytes with the PAXgene Blood System (Becton Dickinson). cDNA was prepared with a Superscript II Reverse Transcriptase Kit (Invitrogen, Carlsbad, CA, USA) and RT-PCR was performed according to the manufacturer’s instructions. Subsequent trio exome sequencing, performed as previously described (Bosch et al. 2021), did not reveal any additional (likely) pathogenic variants in other NDD-associated genes. The *ARID1B* truncating deletion 2188ter in Ind2 was identified in a research setting and shown to be NMD-escaping as described in our previous publications (Hoyer et al. 2012; Vasileiou et al. 2015). An RNA sample was not available. The remaining *ARID1B* missense variants examined in the herein study were collected either from the literature (six) (Mignot et al. 2016; Aref-Eshghi et al. 2018b; Yan et al. 2019; Miyamoto et al. 2021) or ClinVar (two). Detailed information on all variants and their classification is included in the section “Results”, File S1 “variants”, and Table S1.

### RNA-Sequencing

Libraries from Ind1-2129del4, six CSS individuals with *ARID1B* haploinsufficiency variants and nine controls were generated from 0.5 µg high quality RNA using the TruSeq Stranded mRNA Kit (Illumina, San Diego, U.S.A.) according to the manufacturer’s instructions. Libraries were sequenced on a HiSeq 2500 platform (Illumina, San Diego, U.S.A.) as 101 bp single-end reads to a depth of at least 25 million reads. Reads were converted to FASTQ format while masking adapter sequences (bcl2fastq v2.17.1.4, Illumina, San Diego, U.S.A). Low quality bases, poly-A or poly-T stretches and masked regions were trimmed (fqtrim v0.9.5), discarding reads shorter than 50 bp. Data quality was checked after sequencing and after base trimming (fastqc v0.11.7). Samples with more than 15% of reads discarded in the filtering step were excluded. Trimmed reads were mapped to the *Homo sapiens* reference genome GRCh37 and Ensembl gene annotation v85, using a splice-aware aligner (STAR v2.6.1c (Dobin et al. 2013), and quantified as reads per gene while excluding exons shared between more than one gene (samtools v1.8, subread v1.6.1). Based on the gene count matrix, differentially expressed genes were determined using the negative binomial model as implemented in DESeq2 (DESeq2 v1.28.1, R v4.0.2 (Love et al. 2014). Log2 fold changes from highly variable genes were shrunk (apeglm, v1.10.0 (Zhu et al. 2019). Results from significance tests were corrected for multiple testing (Benjamini-Hochberg). For the Heatmap, differentially expressed genes with an abs(logFC) > 2 and a p_adj−_value < 0.01 were used.

### Array-based DNA methylation analysis

Methylation analysis of Ind1-2129del4 and Ind2-2188ter was conducted using the clinically validated EpiSign assay, following previously established methods (Aref-Eshghi et al. 2019, 2020; Sadikovic et al. 2021; Levy et al. 2022). Methylated and unmethylated signal intensities generated from the EPIC array were imported into R 3.5.1 for normalization, background correction, and filtering. Beta values were then calculated as a measure of methylation level, ranging from 0 (no methylation) to 1 (complete methylation), and processed through the established support vector machine (SVM) classification algorithm for EpiSign disorders. The classifier utilized the EpiSign Knowledge Database, which consists of over 10,000 methylation profiles from reference disorder-specific and unaffected control cohorts, to generate disorder-specific methylation variant pathogenicity (MVP) scores. These MVP scores are a measure of prediction confidence for each disorder and range from 0 (discordant) to 1 (highly concordant). A positive classification typically generates MVP scores greater than 0.5. The final matched EpiSign result is generated using these scores, along with the assessment of hierarchical clustering and multidimensional scaling.

### Functional analyses of variants

T7-tagged ARID1B (plasmid #17,987 (Inoue et al. 2002) and FLAG-tagged SMARCA4 (plasmid #19,143 (Xi et al. 2008) were obtained from Addgene. *ARID1B* variants were introduced using the In-Fusion HD Cloning Kit (Clontech). Plasmids were transfected into HEK293T or HeLa cells using JetPrime (Polyplus Life Science). Immunofluorescence staining and protein stability assessments were performed as previously described (Bosch et al. 2023). Co-immunoprecipitation was performed using Dynabeads (Thermo Fisher Scientific). Proximity ligation assay (PLA) was performed using Duolink In Situ Reagents (Sigma). File S2 “Supplementary methods” contains experimental details, oligonucleotide sequences (Table S4), and antibodies (Table S5).

### 3D structural analysis

For the structural analysis, a model of ARID1B generated by AlphaFold (Jumper et al. 2021; Varadi et al. 2022) was used. The effect of the missense variants was assessed with Vipur (Baugh et al. 2016) and AlphaMissense (Cheng et al. 2023). Amylogenic sequence stretches were identified with WALTZ (Maurer-Stroh et al. 2010) using standard settings. RasMol (Sayle 1995) was used for structure visualization.

## Results

### *ARID1B* variants included in the study

Overall, we analysed seven non-truncating variants located in the EHD2 domain of ARID1B (Fig. 1C, Table S1). Variants were annotated to *ARID1B* reference transcript NM_020732.3 (GRCh37/hg19). All variants were absent from gnomAD, with the exception of E2011V (one heterozygous carrier). The novel inframe variant 2129del4 (c.6385_6397delinsA p.(Glu2129_Ala2133delinsThr)) (Fig. 1C) occurred *de novo* in an individual with coarse facial features, mild developmental delay (DD)/intellectual disability (ID), speech deficits, autistic behaviour, muscular hypotonia, complete agenesis of corpus callosum and hydrocephalus internus (Fig. 1A, File S1 “clinical_table”, File S2 “clinical reports”). Coffin-Siris syndrome was clinically suspected. Given its non-truncating nature (as shown by RT-PCR analysis, Figure S1) it was initially classified as variant of unknown significance (VUS; PM2_supporting, PM4_supporting, PS2_supporting). The second variant herein described is a *de novo* frameshift deletion 2188ter (c.6463_6473del p.(Ser2155Leufs*33)), which escapes NMD, leading to the generation of an aberrant transcript (Vasileiou et al. 2015) (Fig. 1C). It was identified in a mildly affected CSS individual (Hoyer et al. 2012) (Fig. 1B, File S1 “clinical_table”). Five additional amino acid substitutions in EHD2 were extracted from the literature (Fig. 1C). Their classification in the respective studies was used. Variant C1945R (c.5833T > C p.(Cys1945Arg)) was identified *de novo* in an individual with clinical suspicion of CSS, and initially classified as VUS. However, *in silico* analysis including evolutionary conservation and protein predictors suggested a deleterious effect, and a methylation assay revealed a BAFopathy episignature (Aref-Eshghi et al. 2018a). Variant I2031N (c.6092T > A p.(Ile2031Asn)) occurred *de novo* in an individual with mild DD/ID and dysplasia with agenesis of the splenium of corpus callosum. *In silico* assessment supported pathogenicity, and it was interpreted as likely pathogenic (Yan et al. 2019). Variant I2031T (c.6092T > C p.(IIe2031Thr)) was reported as causative in a CSS individual with complete agenesis of the corpus callosum and mild DD/ID. It was inherited from the affected mother also presenting with mild ID, but no callosal anomalies (Mignot et al. 2016). In ClinVar it was listed as likely pathogenic. Variant H2054P (c.6161 A > C p.(His2054Pro)) was found *de novo* in an individual with complete agenesis of corpus callosum and mild DD/ID and was classified as likely pathogenic (Miyamoto et al. 2021). The last EHD2 variant E2011V (c.6032 A > T p.(Glu2011Val)) was characterised as VUS. Although no clinical data were provided, the variant did not show a CSS methylation profile (Aref-Eshghi et al. 2018a), and was herein used as a negative control. Available clinical and genetic data of all individuals are described in File S1 “clinical_table” and “variants”.

To exclude any artefacts in functional experiments, we analysed additional variants located outside of the EHD2 domain (Fig. 1C). Two of them were amino acid changes located in the globular ARID domain, sourced from the ClinVar database: D1099V (c.3296 A > T p.(Asp1099Val)) and G1112D (c.3335G > A p.(Gly1112Asp)). They were not observed in gnomAD and *in silico* prediction programmes categorised them as deleterious. Clinical information or inheritance pattern were not available, but both were listed as likely pathogenic. The third variant D1727N (c.5179G > A p.(Asp1727Asn)) lies outside any functional domain and was present in gnomAD (11 heterozygous carriers). It was initially classified as a VUS but subsequently downgraded to likely benign because it did not show a BAFopathy methylation pattern (Aref-Eshghi et al. 2018a) (File S1 “clinical_table” and “variants”, Table S1).

### Expression and methylation profiles are consistent with BAFopathy

**Fig. 2:**
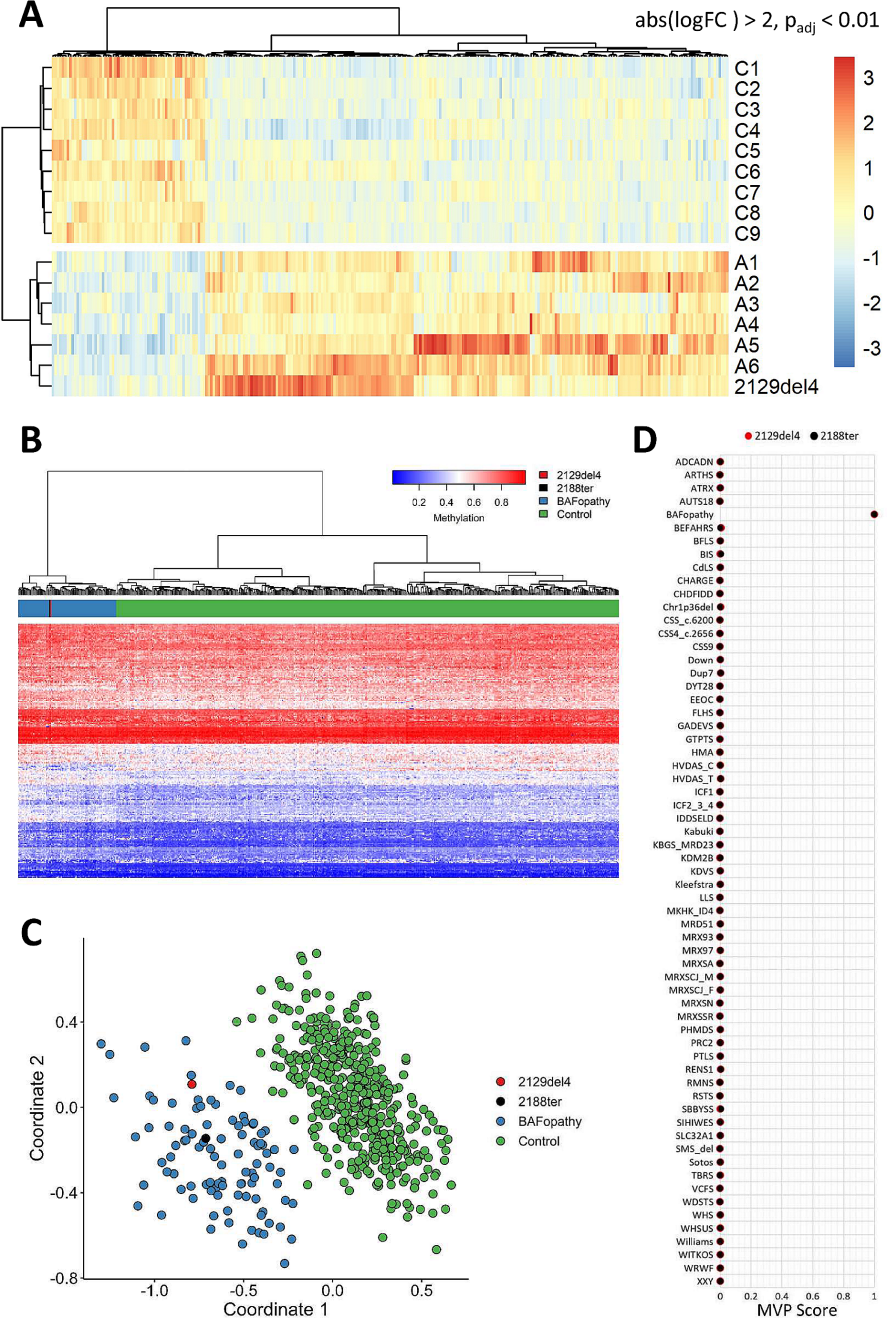
RNAseq and methylation analyses. **(A)** Heat map of differential expression profiles generated from blood of Ind1 (2129del4), six Coffin-Siris (CSS) individuals with truncating variants in *ARID1B* (A1-A6) and nine controls (C1-C9). Gene expression is scaled across columns. Note that the expression pattern of Ind1 clusters together with CSS individuals and separately from controls. **(B-D)** EpiSign (DNA methylation) analysis in peripheral blood from two cases with variants 2129del4 and 2188ter in *ARID1B*. **(B)** Hierarchical clustering and **(C)** multidimensional scaling plots indicate that lnd1 (2129del4) (red) and lnd2 (2188ter) (black) both have a DNA methylation profile similar to subjects with a confirmed BAFopathy episignature (blue) and distinct from controls (green). **(D)** MVP score, a multi-class supervised classification system capable of discerning between multiple episignatures by generating a probability score for each episignature. The BAFopathy score of 1.0 for both cases indicates an episignature similar to BAFopathy reference cases, including those with Coffin-Siris syndrome 1

Despite the initial classification of the indel variant 2129del4 as VUS, the strong resemblance of the individual´s presentation to CSS required further investigation. To examine a potential clinical significance, we performed transcriptome analysis, comparing its expression profile to six CSS individuals harbouring pathogenic NMD-inducing *ARID1B* variants and nine healthy controls. We observed that its expression pattern clustered together with that of the *ARID1B* truncating alterations, and was distinct from that of controls (Fig. 2A). An RNA sample for testing of the 2188ter deletion was not available. Nevertheless, a previous transcriptome analysis including this variant revealed a similar clustering with pathogenic NMD-inducing *ARID1B* variants (Vasileiou et al. 2015). Additionally, array-based DNA methylation analysis upon EpiSign assay was applied to samples of both individuals and revealed a genome-wide DNA methylation profile consistent with BAFopathy syndromes (Fig. 2B-D). More specifically, as indicated by Euclidean clustering, multidimensional scaling and an elevated MVP score (both cases = 1.0), the methylation signatures of both the inframe insertion-deletion and frameshift deletion individuals were concordant with those observed in individuals with *ARID1A*, *ARID1B*, *SMARCB1*, *SMARCA4* and *SMARCA2* variants.

### EHD2 variants do not generally impact the interaction with SMARCA4

ARID1B interacts with SMARCA4 via its EHD2 domain (Inoue et al. 2002). Interestingly, it has previously been shown that the NMD-escaping frameshift variant 2188ter leads to weaker interaction with SMARCA4 (Vasileiou et al. 2015). Considering an impaired interaction with SMARCA4 as plausible cause of pathogenicity, we explored if this was also the case for other EHD2 variants. To this end, ARID1B-T7 expression vectors harbouring the different EHD2 domain variants were generated. We overexpressed the vectors together with SMARCA4-FLAG in HEK293T cells and analysed the interaction through proximity ligation (PLA) as well as co-immunoprecipitation assays (CoIP). While the PLA showed qualitative interaction of all tested ARID1B variants with SMARCA4 (Fig. S2A), quantitative CoIP confirmed that this interaction was indeed markedly reduced for the frameshift variant 2188ter. No effect was shown for the remaining EHD2 variants (Fig. S2B).

### Variants in the EHD2 and ARID domains are prone to misfolding and aggregation

**Fig. 3:**
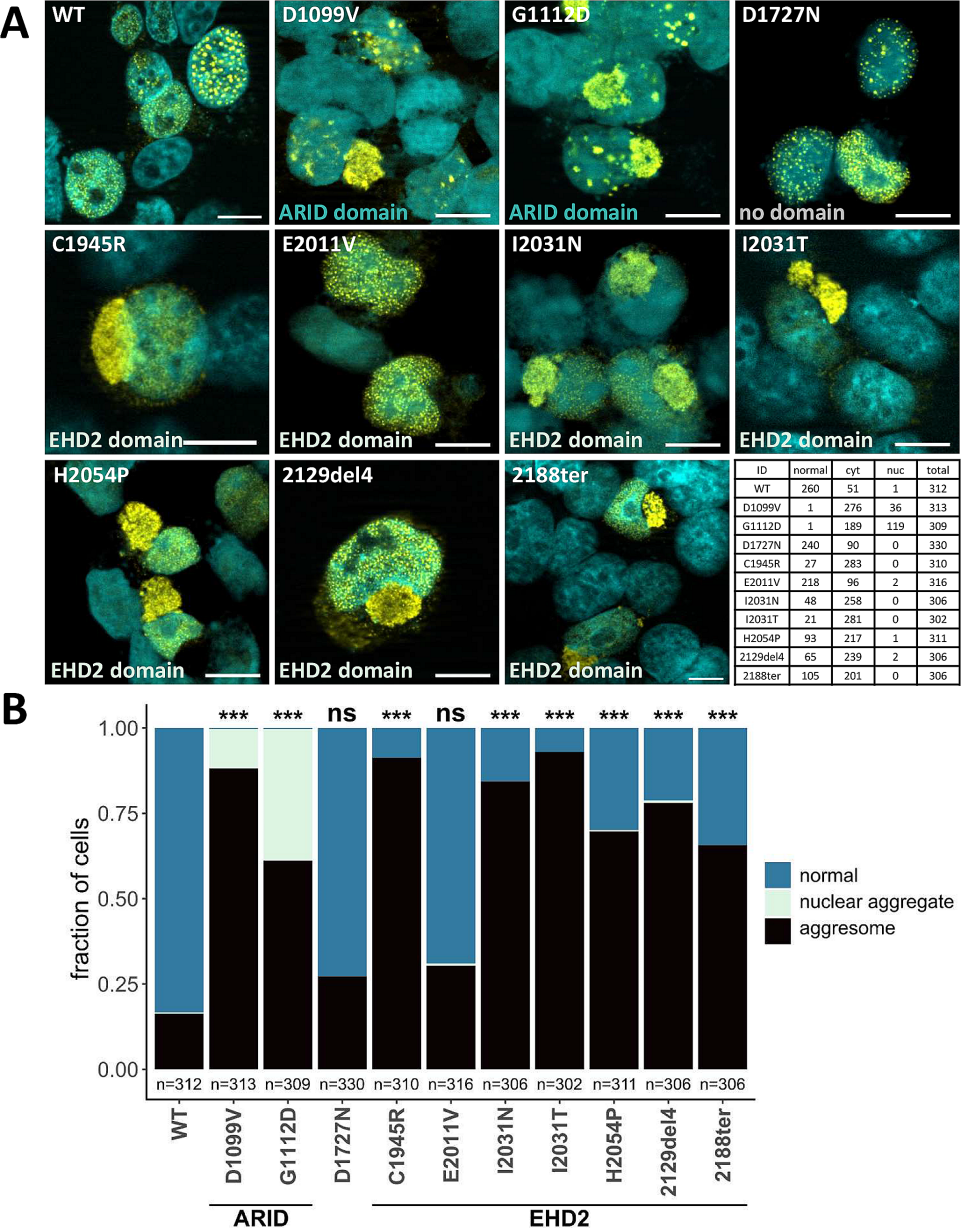
Immunofluorescence analysis shows protein aggregation. **(A)** Representative microscopy images of intracellular localization of ARID1B wild type (WT) and mutants overexpressed in HEK293T cells. Scale bar: 10 μm. WT ARID1B is distributed either evenly throughout the nucleus or in nuclear puncta. Variants in the ARID domain (D1099V, G1112D) exhibit cytoplasmic as well as nuclear aggregation. Variant D1727N located outside of any functional domain as well as the EHD2 variant E2011V show normal distribution, whereas all remaining EHD2 variants aggregate in the cytoplasm. Cell counts for quantification are shown in a contingency table. **(B)** Quantification of aggregation. Statistical analysis: in three independent experiments, 100 transfected cells each were analyzed for ARID1B localization. Bars show the fraction of cells with the respective localization pattern: normal (blue), nuclear aggregation (light green), and cytoplasmic aggregation (black). P-values were generated using a chi-squared test and corrected for multiple testing. Significant aggregation compared to the wild type was observed for all variants except D1727N and E2011V. *** *p* < 0.001

As amino acid substitutions and NMD-escaping deletions can affect protein folding and structure, we addressed whether this holds true for variants in the EHD2 domain of ARID1B. To this end, the subcellular localization was examined upon overexpression in HEK293T cells via immunofluorescence staining. Depending on the cell cycle, ARID1B was either homogeneously distributed or in a punctate pattern throughout the nucleus (Vasileiou et al. 2015) (Fig. 3A, wild type; WT). Four of the five EHD2 missense (C1945R, I2031T, I2031N, H2054P) as well as the indel and frameshift variants predominantly showed protein accumulation in circular cytoplasmic formations in 66–93% of the examined cells, depending on the variant. Such formations were only observed in 16% of cells expressing wild type protein, most likely as a result of cellular protein overload due to overexpression (Fig. 3A-B). The EHD2 missense variant E2011V and the variant D1727N lying outside of known functional domains did not show significantly increased formation of cytoplasmic aggregation, with only ~ 30% of observed cells affected (Fig. 3A-B). Surprisingly, the aggregation was more pronounced for the two ARID substitutions (D1099V, G1112D), which exhibited not only cytoplasmic aggregates (in 61 to 88% of cells), but also smaller, nuclear aggregates (12% and 39%). As a result, less than 1% of observed cells displayed the normal nuclear ARID1B distribution (Fig. 3A-B).

**Fig. 4:**
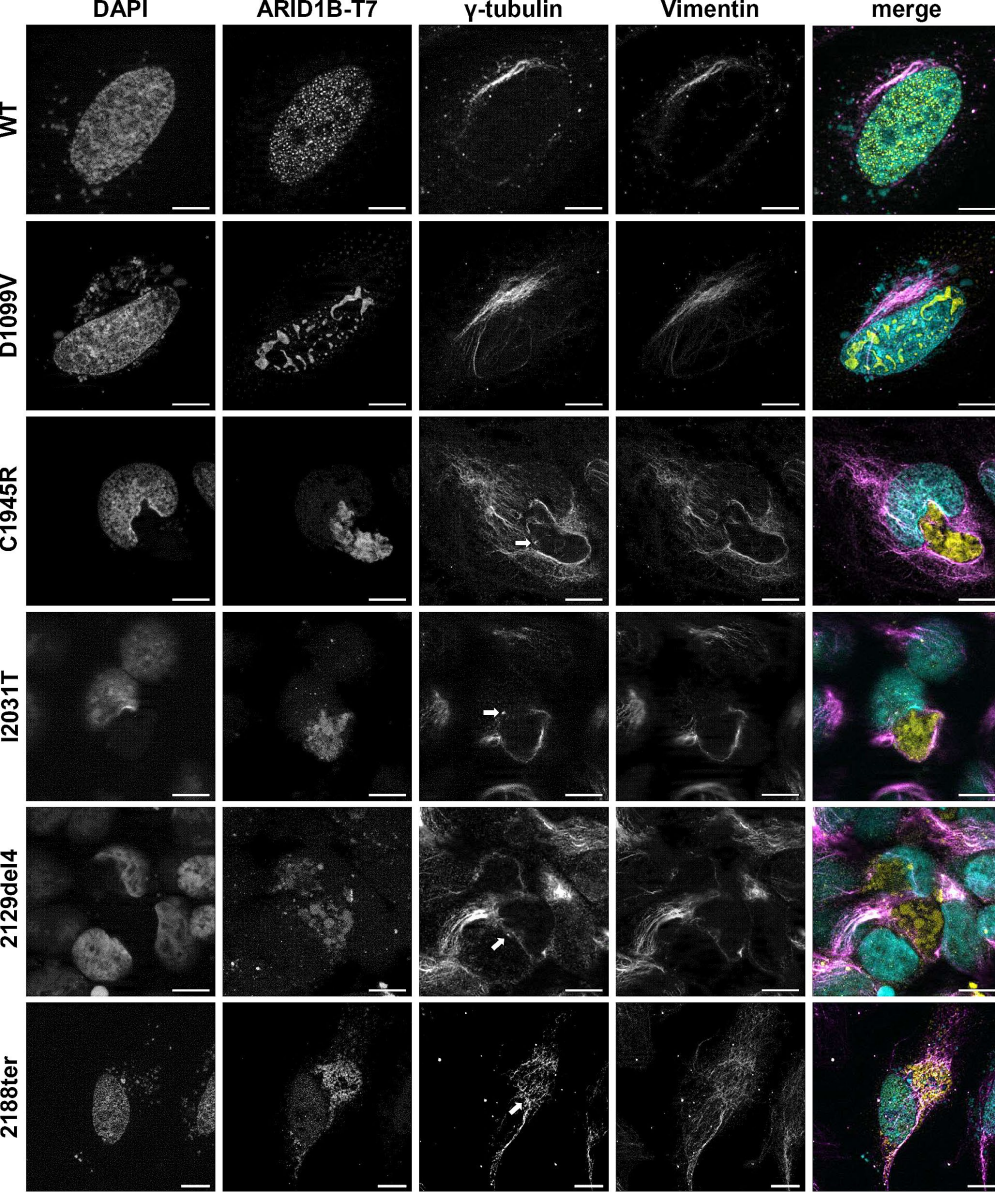
Identification of aggregates as aggresomes. Co-staining of ARID1B WT and variant proteins with the cytoskeletal filament protein vimentin and the centromere protein γ-tubulin shows inclusion of aggresomes in a vimentin cage and close proximity to the MTOC, indicated by arrows. Scale bar: 10 μm

The cytoplasmic aggregates resembled structures previously described as aggresomes. These are juxtanuclear inclusion bodies in close proximity to the microtubule organisation centre (MTOC), and are surrounded by the intermediate filament protein vimentin (Johnston et al. 1998; Johnston and Samant 2021). A co-staining of transfected HeLa cells with vimentin and *γ*-tubulin (centromere marker), revealed both the characteristic vimentin cage-like structure around the cytoplasmic formations as well as a co-localisation with the MTOC, further confirming our hypothesis (Fig. 4, Fig. S3).

Furthermore, except for the ARID variant D1099V that showed significantly reduced protein expression, the total protein levels were comparable between wild type and protein variants according to western blot analysis (Fig. S4).

### Aggregation is likely caused by exposure of amylogenic protein stretches

**Fig. 5:**
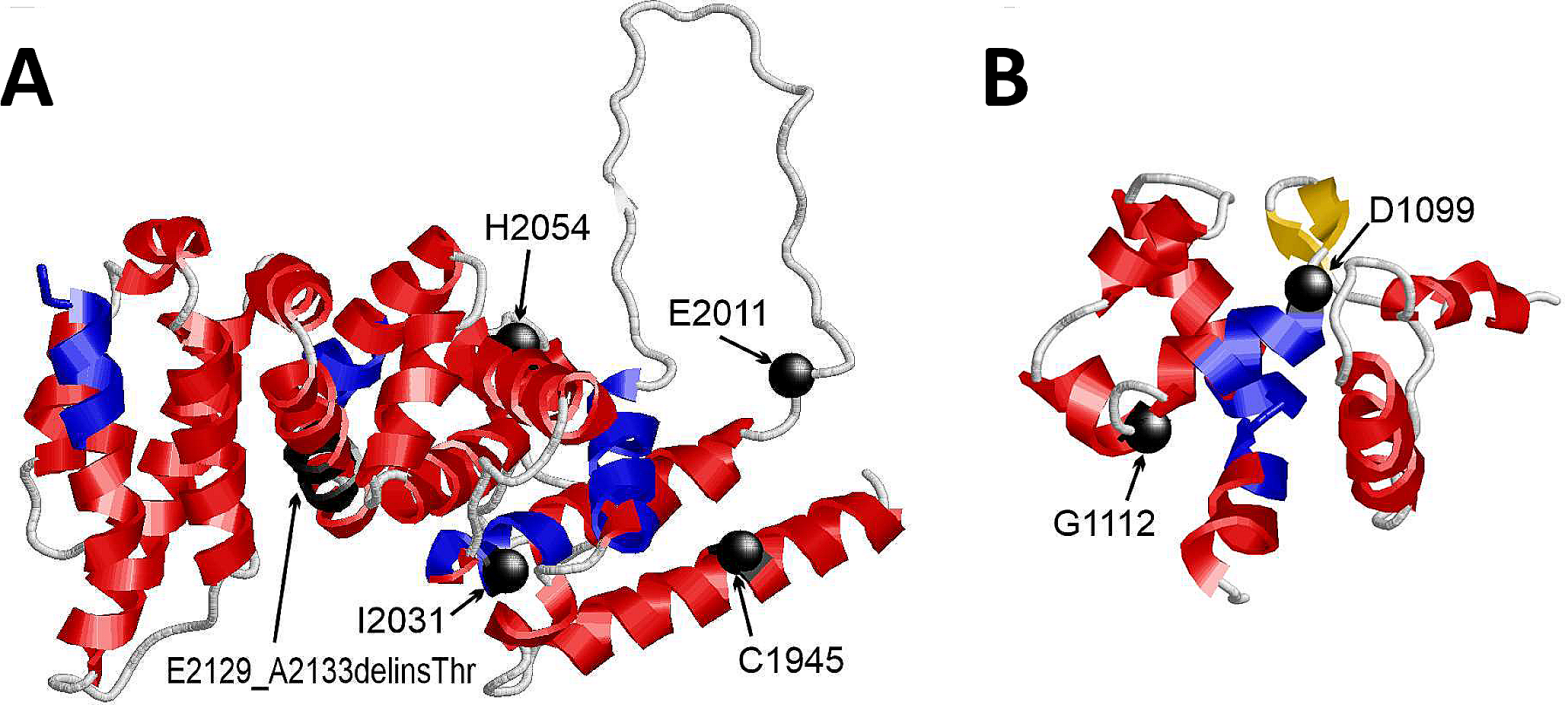
Structural analysis of ARID1B variants. **(A)** Structure of the EHD2 domain indicating the sites of mutation as black balls. The stretch of the E2129_A2133delinsThr mutation is highlighted as black ribbon. The amylogenic sequence stretches are marked in blue. **(B)** Structure of the ARID domain indicating the sites of mutation as black balls. The amylogenic sequence stretches are marked in blue

Computational analysis showed that the EHD2-domain exhibits amylogenic sequences (Fig. 5A, Table S2). The four aggregating missense variants (C1945R, I2031T, I2031N, H2054P) are located in the globular part of the EHD2 domain near the amylogenic segments. Since these variants are predicted to severely disrupt the domain structure (Table S3), the amylogenic sequence stretches will get exposed, thereby likely leading to protein aggregation (Teng and Eisenberg 2009). A similar mode of action is likely for the 2129del4 and 2188ter variants, which are predicted to cause an entire loss of the three-dimensional EHD2 domain structure.

The ARID missense variants (D1099V, G1112D) were also predicted to be deleterious according to the AlphaMissense and Vipur predictions (Table S3). They are flanking a sequence stretch (L1100-V1105), which is predicted to exhibit amylogenic properties (Fig. 5B, Table S2). Similar to the EHD2 variants, the two substitutions in the ARID domain are expected to disrupt the three-dimensional structure, thereby offering an explanation for the experimentally observed aggregation.

The two remaining missense alterations (E2011V, D1727N) showed no significantly increased aggregation in the functional assays confirming their initial classification as not causative. This property most likely results from their location within the ARID1B structure. Variant E2011V is located in a long disordered loop of the EHD2 domain (Fig. 5A). Therefore, the effect of the exchange is likely less severe compared to those variants in the globular part of the EHD2 domain. Variant D1727N is located outside of the globular domains (Fig. 5A), so that the exchange is not expected to have a critical impact on ARID1B structure and aggregation properties.

## Discussion

Here we present compelling evidence for the pathogenicity of non-truncating or NMD-escaping variants in the EHD2 and ARID functional domains of the *ARID1B* gene, thereby providing novel insights into the understanding of *ARID1B*-associated CSS with implications for genetic diagnosis.

We initially employed gene expression profiling and subsequent methylation analysis from peripheral blood samples to confirm the clinical relevance of variant 2129del4 in Ind1. Both methods reached the same outcome, reliably classifying Ind1 in the ARID1B-CSS group. The previously performed transcriptome (Vasileiou et al. 2015) and herein examined methylation analysis of the frameshift deletion 2188ter showed similar results. One additional alteration, the previously investigated variant C1945R (Aref-Eshghi et al. 2018a), also revealed a BAFopathy methylation profile, whereas two others (E2011V, D1727N (Aref-Eshghi et al. 2018a) did not. In all cases, our experimental findings confirmed the results of RNA-Seq and DNA-methylation episignatures, both of which have rapidly found their way into research and diagnostic contexts (Stenton and Prokisch 2020; Sadikovic et al. 2021). Taken together, both approaches can reliably be used for the classification of non-truncating *ARID1B* variants.

ARID1A/B subunits interface with SMARCA4 via their conserved EHD2 domain. SMARCA4 missense variants that impair the interaction with the EHD2 domain of the ARID1B paralog, ARID1A, have previously been linked to reduced BAF complex function (Mashtalir et al. 2020). Based on these data, compromised interaction with SMARCA4 was presumed initially as the mechanism of pathogenicity for EHD2 non-truncating variants in ARID1B. However, decreased interaction was observed only for the frameshift variant 2188ter, which deletes 61 amino acids from the EHD2 domain (roughly 10%), thus having the largest impact on the overall structure. These results indicate that loss of interaction with SMARCA4 is not the main cause of pathogenicity for EHD2 variants. This conclusion is in accordance with a systematic mutational screen, finding only few EHD2 variants with the potential to impact binding to SMARCA4 (Mermet-Meillon et al. 2024).

Instead, we observed the formation of aggresomes in the majority of EHD2 and both ARID variants. The only alterations that did not exhibit aggregation were the two variants which did not show a BAFopathy methylation profile (D1727N, E2011V). Aggresomes, also known as microtubule-dependent cytoplasmic inclusion bodies, are pericentriolar structures owing their extreme stability to the surrounding vimentin cage. They arise when proteasome capacity is exceeded by an overload of misfolded, mostly poly-ubiquitinated proteins, subsequently leading to the accumulation of peripheral small protein aggregates proximal to the MTOC (Johnston et al. 1998; Ajmal 2023). Nuclear aggregates like the ones observed in ARID domain variants have also been associated with an abnormal protein conformation (Ajmal 2023). By measuring protein levels in the cell through a stability sensor assay, Mermet-Meillon and colleagues described a negative effect of EHD2 missense variants on protein stability. Nevertheless, the applied methodology could not differentiate between protein misfolding, destabilisation or mislocalisation (Mermet-Meillon et al. 2024). Our findings further elucidate the pathomechanism of the EHD2 non-truncating variants by revealing a loss-of-protein function due to misfolding and aggregation. Amino acid substitutions in the ARID domain exhibited the same effect. The loss-of-function pathomechanism of the non-truncating or NMD-escaping variants in EHD2 and ARID domains is further supported by the indistinguishable clinical presentation of their carriers and those harbouring pathogenic *ARID1B* NMD-inducing variants. The formation of aggregation is most likely attributed to the exposure of amylogenic protein stretches of the EHD2 and ARID domain due to the nearby alterations (Teng and Eisenberg 2009). Interestingly, the amylogenic segments (Fig. 5 and Table S2) are part of the central helical structures within the EHD2 domain, which were previously reported to be particularly sensitive to pathogenic variants (Mermet-Meillon et al. 2024).

Furthermore, Mermet-Meillon and colleagues showed that some clinically relevant EHD2 variants from ClinVar caused decreased ARID1B protein levels (Mermet-Meillon et al. 2024). Their analysis included the missense variant I2031T, referred to as I2018T under their nomenclature, which was also examined in our study. The authors concluded that I2031T would lead to reduced protein levels, according to a FACS-based assay. On the contrary, quantitative western blot analysis in the present study showed that all aggregating EHD2 variants, including I2031T, exhibit the same ARID1B protein levels as the wild type protein (Fig. S4). The difference between the two studies may likely be attributed to the different methodologies applied. Specifically, our study addressed protein levels of the entire wild type protein or I2031T variant, whereas the aforementioned study specifically addressed stability of the EHD2 domain fragment. Moreover, we demonstrated the formation of aggresomes, which are considered stable formations, possibly leading to the preservation of total protein levels. The aggregating ARID variant D1099V was the only alteration that exhibited a reduction in protein stability. This result might indicate a more complex molecular pathomechanism for this specific variant that requires further investigation.

The *ARID1B*-associated BAFopathy belongs to the mild CSS spectrum. Nevertheless, moderate and severe CSS cases have also been described (Hoyer et al. 2012; van der Sluijs et al. 2019; Vasko et al. 2021; Schmetz et al. 2024). Five of the herein described individuals with aggregating EHD2 variants and available clinical description as well as the carrier mother of the individual harboring the I2031T variant presented with mild DD/ID. No other serious malformations or congenital anomalies were noted (Mignot et al. 2016; Yan et al. 2019; Miyamoto et al. 2021). Four of them displayed corpus callosum agenesis (File S1 “clinical_table”, File S2 “clinical reports”). In the literature one additional *de novo* (likely) pathogenic EHD2 missense change (c.5855T > C p.(Met1952Thr)) in an individual with mild ID, epilepsy and marfanoid features has been reported (Chevarin et al. 2020). Unfortunately, ClinVar entries lack the corresponding clinical information. Although the initial clinical descriptions point to a mild CSS phenotype, a conclusive assessment concerning phenotypic severity requires a larger cohort of CSS cases with non-truncating EHD2 variants. For alterations in the ARID domain there is no clinical information reported to date.

So far, the pathogenicity of non-truncating *ARID1B* variants was controversial due to the lack of experimental data, as reflected in the respective ClinVar entries. Indeed, many of the listed EHD2 and ARID domain variants are interpreted as VUS, whereas some alterations outside of these domains have been classified as (likely) pathogenic without functional evidence. Our study together with the recently published findings of Mermet-Meillon and colleagues (Mermet-Meillon et al. 2024) experimentally confirms the pathogenicity of non-truncating or NMD-escaping EHD2 variants. To our knowledge, the effect of non-truncating ARID domain variants has not been previously investigated. Nevertheless, the functional studies presented here demonstrate robust pathological effects. We understand that missense variants are difficult to interpret and that not all EHD2 or ARID domain alterations will be causative. Structural predictions, like the AlphaMissense score, and methylation or transcriptome analysis constitute reliable supporting tools for further clarification, yet they are not always available in everyday practice. To enable an effective assessment in the diagnostic setting we propose that *de novo* variants (PS2_supporting) in the EHD2 or ARID domains (PM1_moderate), which are absent or infrequent (≤ 2) in population databases (PM2_supporting) and reach computational evidence thresholds (PP3_supporting or PP3 moderate) in individuals with CSS clinical features can be a priori interpreted as likely pathogenic.

In conclusion, we demonstrate the pathogenicity of yet unexplored non-truncating/NMD-escaping variants in the EHD2 domain of ARID1B. Additionally our study suggests the causality of variants located in the ARID functional domain of ARID1B. Experimental investigation reveals for the first time the underlying pathomechanism for both EHD2 and ARID domain variants, which involves protein accumulation in stable aggresomes or nuclear aggregates due to protein misfolding. Consequently, our findings set the starting point for (re-)evaluation of unclear non-truncating changes in the *ARID1B* gene.

### Electronic supplementary material

Below is the link to the electronic supplementary material.

Supplementary Material 1: File S1 Excel file containing the worksheet “clinical_table” with comprehensive clinical and genetic data of individuals included in the herein study, the worksheets “domains” and “variants” used for Fig. 1C, as well as the worksheet “missense_clinvar” containing pathogenic/likely pathogenic missense variants listed in the ClinVar database

Supplementary Material 2: File S2 Supplementary notes with clinical reports of Ind1 and Ind2, supplementary methods, figures, tables, and references

## Data Availability

The data supporting this article are provided in the supplementary files available in the online version of this article at the publisher’s website.
