# Supplementary material for: The missing link: ARID1B non-truncating variants causing Coffin-Siris syndrome due to protein aggregation
Source: Hum Genet. 2024 Jul 19;143(8):965–78. doi: 10.1007/s00439-024-02688-9 (PMC11303441; doi:10.1007/s00439-024-02688-9)
Supplement: Supplementary file 2 — Supplementary Material 2: File S2 Supplementary notes with clinical reports of Ind1 and Ind2, supplementary methods, figures, tables, and references [file 439_2024_2688_MOESM2_ESM.pdf]

SUPPLEMENTARY DATA

The missing link: *ARID1B* non-truncating variants causing Coffin-Siris syndrome due to protein aggregation

Elisabeth Bosch[1], Esther Güse[1], Philipp Kirchner[1], Andreas Winterpacht[1], Mona Walther[1], Marielle Alders[2], Jennifer Kerkhof[3], Arif B. Ekici[1], Heinrich Sticht[4], Bekim Sadikovic[3,5], André Reis[1,6], Georgia Vasileiou*[1,6]

CLINICAL REPORTS

Individual 1 (Ind1-2129del4)

Female individual 1 is the first child of a non-consanguineous family originating from Germany. In prenatal ultrasound agenesis of corpus callosum was suspected. She was born by caesarean section at 39+6 weeks of gestation due to pathological cardiotocography and green staining of amniotic fluid. At second postnatal check-up, individual 1 had a weight of 2840 g (-1.46 SD), a length of 48 cm (-1.64 SD) and an occipitofrontal head circumference (OFC) of 36 cm (+0.88 SD). Postnatally she exhibited a stridor resulting from chondromalacia of the epiglottis. Cranial ultrasound revealed agenesis of corpus callosum and hydrocephalus internus. Gross motor development was mildly impaired, she walked independently with 33 months. Muscular hypotonia and a tendency to overstretching were also observed. Individual 1 spoke her first words at the age of 3 years. At last clinical assessment at 4 years and 7 months, individual 1 presented with a weight of 15.8 kg (-0.94 SD), a height of 101 cm (-1.57 SD) and an OFC of 51.3 cm (+0.7 SD). She could speak in three word sentences and receptive speech was restricted. Gross motor skills were normal. Furthermore, she displayed autistic behaviour. She was initially enrolled in an inclusive programme of a regular kindergarten, but later she changed in a kindergarten for children with special needs. She received early support, physical therapy and speech therapy. Cognitive test and brain MRI have not been performed yet. Apart from an umbilical hernia no further physical anomalies were reported. Facial dysmorphic features included coarse face, periorbital fullness, thick eyebrows, depressed nasal bridge, wide nose, upturned nasal tip, a broad nasal tip, short philtrum, wide mouth, thin upper lip vermilion, everted lower lip vermilion, large, posteriorly rotated ears. She also displayed hypertrichosis at the back, a small hypomelanotic area at the left lower leg and broad big toes.

Both parents were healthy. A 28-year-old maternal cousin was diagnosed with developmental delay.

Individual 2 (Ind2-2188ter)

Male individual 2 is the first child of a non-consanguineous family originating from Germany. The pregnancy was uneventful. He was born at 42 weeks of gestation via caesarean section with a birth weight of 3500 g (-0.64 SD), a length of 52 cm (-0.58 SD) and an OFC of 36 cm (-0.07 SD). Apgar score was 10/10. After birth, hypoxia and a median cleft palate leading to feeding difficulties were detected, the latter was surgically corrected. Additionally, he exhibited muscular hypotonia, therefore he stayed at the hospital for 34 days after birth. He was able to walk independently at the age of 36 months. At clinical assessment at 7 years and 3 months, individual 2 presented with a weight of 21.3 kg (-1.06 SD), a height of 114.5 cm (-2.05 SD) and an OFC of 52 cm (-0.51 SD). He was not able to speak and he was communicating via gestures. Gross motor development was mildly impaired with uncoordinated walking and difficulties in jumping. Behavioral anomalies and epilepsy were not present. Individual 2 was enrolled in a special school. He had a profound myopia (-14/-11 dpt) and nystagmus. Other diagnoses included surgically treated undescended testicles, inguinal hernia and a ventricular septal defect (VSD) that spontaneously closed. Cranial magnetic resonance imaging (cMRI) showed signs of delayed myelinisation. Facial dysmorphic features included sparse hair, down-slanting palpebral fissures, thick and medially fanned out eyebrows, long eyelashes, prominent columella, smooth philtrum, thin upper lip, everted lower lip vermilion, small teeth and posteriorly rotated, slightly protruding, simplified ears. Clinical examination showed bilateral

clinodactyly of the 5th finger, broad big toes with hypoplastic nails, sandal gab left. At the re-evaluation at the age of 19 years and 9 months, his weight was 58 kg (-1.5 SD), height 171 cm (-1.41 SD) and OFC 57 cm (-0.01 SD). He was still not able to speak but communicated via sounding, icons and gestures. Receptive language was restricted. Gross motor deficits were not reported. He was able to hold a pen and write his name. A cognitive test at the age of 16 years showed an intelligence quotient (IQ) of 52. Myopia had worsened (up to -17 dpt). His mouth was often open and he was producing much saliva. Malpositioned teeth and retrognathia were also observed. Sleeping difficulties were present.

Both parents and his sibling were healthy.

SUPPLEMENTARY METHODS

Cell Culture

HEK293T and HeLa cells were grown in Dulbecco's Modified Eagle's Medium (DMEM) supplemented with 10% fetal calf serum (FCS) and 1% penicillin/streptomycin at 37°C in 5% CO2. Plasmids were transfected with JetPrime (Polyplus Life Science) according to manufacturer's instructions.

Plasmids and Mutagenesis

T7-tagged ARID1B was obtained from addgene (plasmid #17987) and has previously been described(Inoue et al. 2002). Mutagenesis was carried out using the In-Fusion HD Cloning kit (Clontech). FLAG-tagged SMARCA4 was obtained from addgene (plasmid #19143)(Xi et al. 2008). Oligonucleotides used for mutagenesis are listed in Table S4.

Immunofluorescence, PLA and Microscopy

Cells were grown on coverslips. 24 h post- transfection, they were fixed in 3.7 % formaldehyde for 10 min at room temperature, permeabilised for 10 min in 0.5 % Triton-X-100 and washed in PBS. Antibodies were diluted in PBS containing 5 % normal goat serum. Cells were incubated with the primary antibodies for 1 h at 37 °C and washed in PBS containing 0.1 % Tween20, followed by incubation with the Alexa Fluor 488-conjugated secondary antibody for 1 h at 37 °C and another wash. Coverslips were mounted using ProLong Antifade mountant (Thermo Fisher Scientific).

For PLA, cells were fixed and permeabilized as described above 30 h post transfection. PLA was performed using Duolink In Situ reagents (Sigma) according to the manufacturer's instructions.

All antibodies used are listed in Table S5.

All slides were imaged on a Zeiss AxioImager Z2 with Apotome using the AxioVision software and processed using AxioVision and ImageJ.

Statistical analysis: 100 cells each from three independent experiments were analysed for the subcellular localisation of ARID1B and statistical significance was calculated using a chi-squared test. To correct for the effects of low contingency values in the chi-squared test, data were deliberately shifted towards the null hypothesis expectation by adding 10% of the overall sum of every contingency table to each cell of that table, so that, for each table with counts $c_1 \dots c_6$, we get a corrected table with counts $c'_1 \dots c'_6$ by applying $c'_\alpha = c_\alpha + \frac{\sum_{\alpha=1}^{6} c_\alpha}{10}$. After conducting all chi-squared tests, p-values were additionally Bonferroni corrected for 10 tests.

Immunoprecipitation and Western Blotting

Whole cell lysate was generated as previously described (Wittmann et al. 2021): Briefly, cells were washed in PBS and rotated in buffer A (10 mM HEPES, pH 7.9; 10 mM KCl; 0.1 mM EDTA, pH 8.0; 0.1 mM EGTA, pH 8.0; 2 mM DTT; 10 µg/ml Aprotinin; 10 µg/ml Leupeptin; 1 % NP-40; 300 mM NaCl) for 15 min at 4 °C. Following centrifugation at 16000 x g for 5 min, protein concentration was determined using the Qubit Protein Assay-kit (Thermo Fisher Scientific).

Co-immunoprecipitation of FLAG-tagged SMARCA4 was performed using magnetic Dynabeads (Thermo Fisher Scientific) without crosslinking according to manufacturer's instruction. After conjugation of 2 µg of antibody (mouse monoclonal FLAG, Sigma F1804) per 50 µl of magnetic beads, 3 mg of whole cell lysate was added and rotated for 10 min at room temperature. Following three washes in PBS, proteins were eluted in 50 µl 2x Lämmli-buffer at 70 °C for 10 minutes.

Protein stability was assessed by transiently co-expressing T7-tagged ARID1B variants together with a different-sized control protein (HA-tagged TBX1), followed by quantitative western blot analysis and normalization of ARID1B-T7 to TBX1-HA. Histone H3 was stained as a loading control.

10 µl of the IP fraction or 1.5 mg of total protein lysate were run on 4-15 % gradient polyacrylamide gels (BioRad) and transferred onto nitrocellulose membranes using semi-dry blotting. Membranes were blocked in 5 % non-fat dry milk in TBS for 1 h at room temperature and incubated with antibodies in 3 % non-fat dry milk in TBS-T. Incubation with primary antibodies occurred overnight at 4 °C. Following three washes in TBS-T, secondary antibodies were incubated for 1 h at room temperature. Membranes were developed with SuperSignal™ West Femto Maximum Sensitivity Substrate (Thermo Scientific) and imaged on a ChemiDoc Imaging System (BioRad). Protein bands were quantified by densitometry using the software ImageLab (BioRad).

All antibodies used are listed in Table S5.

Statistical analysis for CoIP: Data was generated from three independent experiments. P-values were calculated using a one sample t-test (hypothetical mean = 1, significance threshold < 0.05). Statistical analysis for protein stability: The value of wildtype ARID1B-T7 was set to 1. Data from five independent experiments was analysed. P-values were calculated using a one sample t-test (hypothetical mean = 1, significance threshold < 0.05).

SUPPLEMENTARY FIGURES

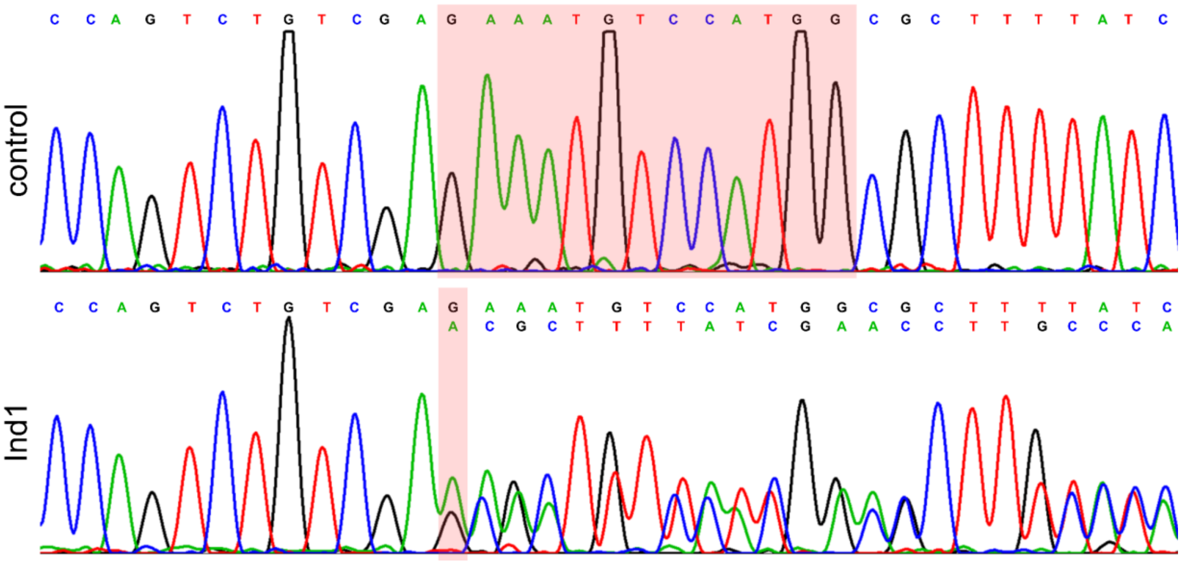

Fig. S1 | RT-PCR-Seq analysis of Ind1

Sanger sequencing of RT-PCR products of a control individual (top) and Ind-1 (bottom) amplified using total RNA extracted from peripheral blood. The insertion-deletion is marked in red.

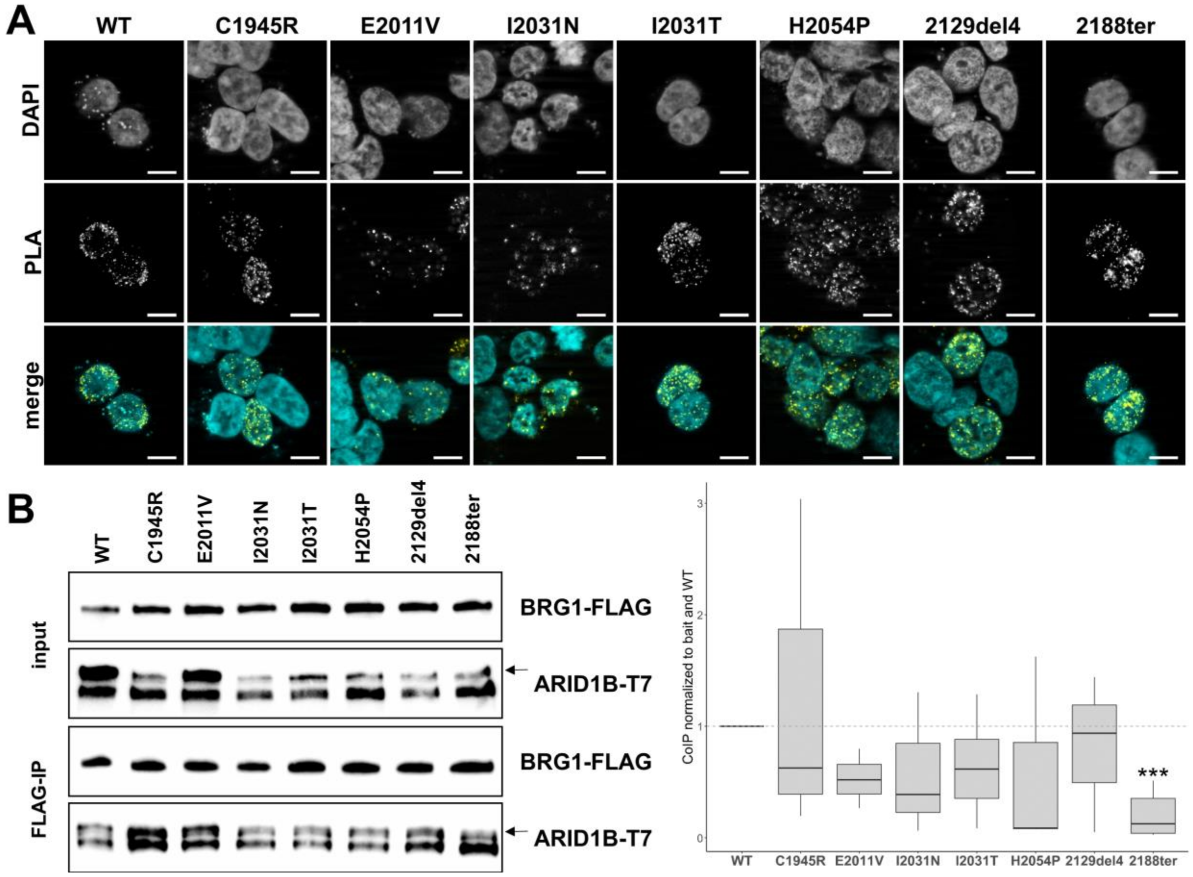

Fig. S2 | ARID1B EHD2 variants do not generally impact SMARCA4 binding

(A) Proximity ligation assay (PLA) of HEK293T cells with transiently overexpressed FLAG-SMARCA4 together with wild type (WT) and mutant T7-tagged ARID1B. yellow: positive PLA signal indicating interaction; blue: DAPI; scale bar: 10 µm. **(B)** Co-Immunoprecipitation (CoIP) of FLAG-SMARCA4. Left panel: representative western blot images of the bait signal (SMARCA4-FLAG) and pulldowns of wild type or mutant ARID1B-T7. Right panel: box plots of signal quantification normalized to the bait protein (SMARCA4-FLAG) and wild type sample. Data generated from at least three independent experiments. P-values were calculated using a one sample t-test (hypothetical mean = 1, significance threshold < 0.05). *** p < 0.001. Note significant loss of interaction between SMARCA4 and truncated ARID1B 2188ter.

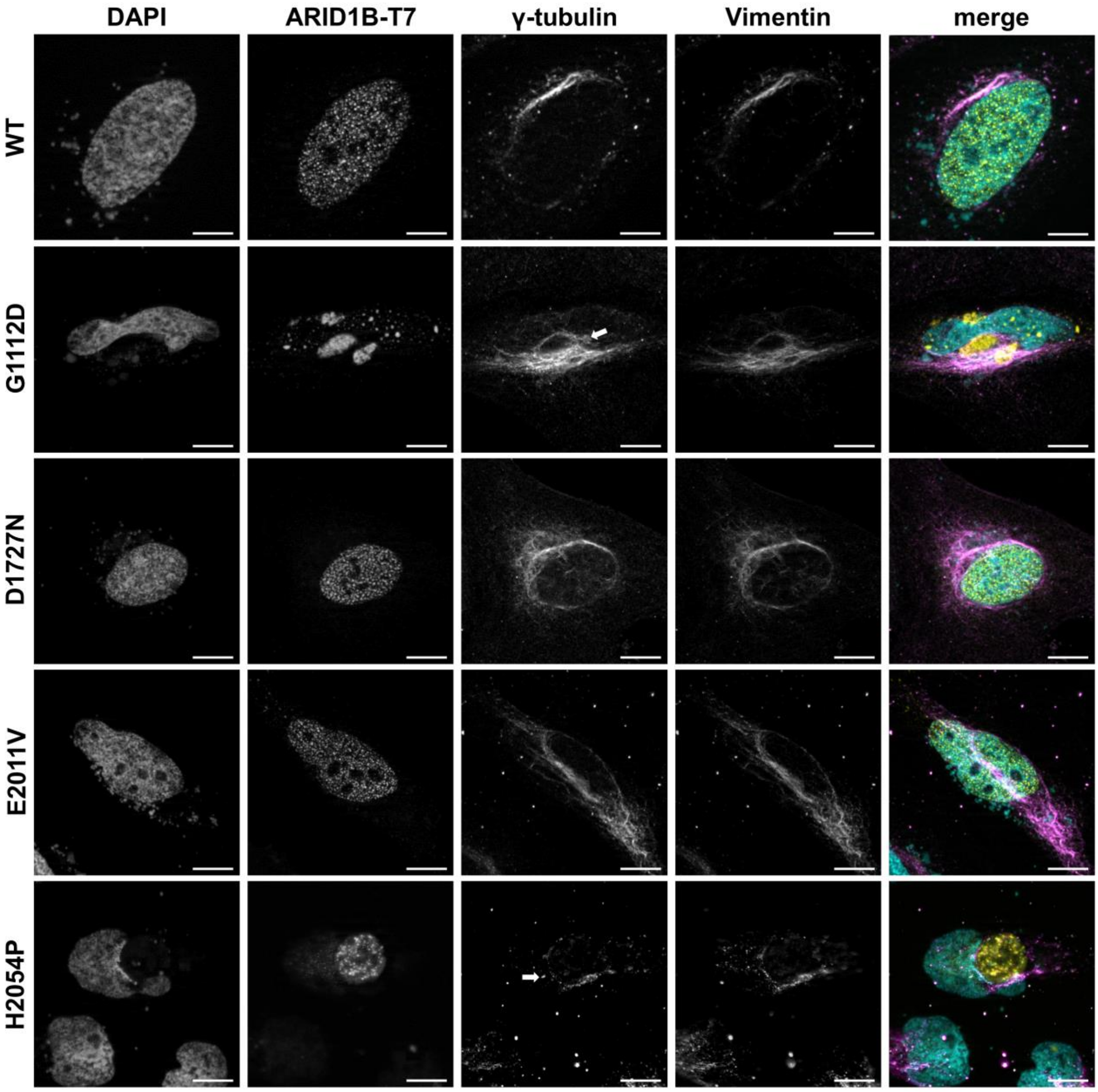

Fig. S3 | Immunofluorescence staining of aggresomes

Co-staining of overexpressed ARID1B WT or variant protein in HeLa cells with the cytoskeletal filament protein vimentin and the centromere protein γ-tubulin shows inclusion of aggresomes in a vimentin cage and close proximity to the MTOC. Scale bar: 10 µm.

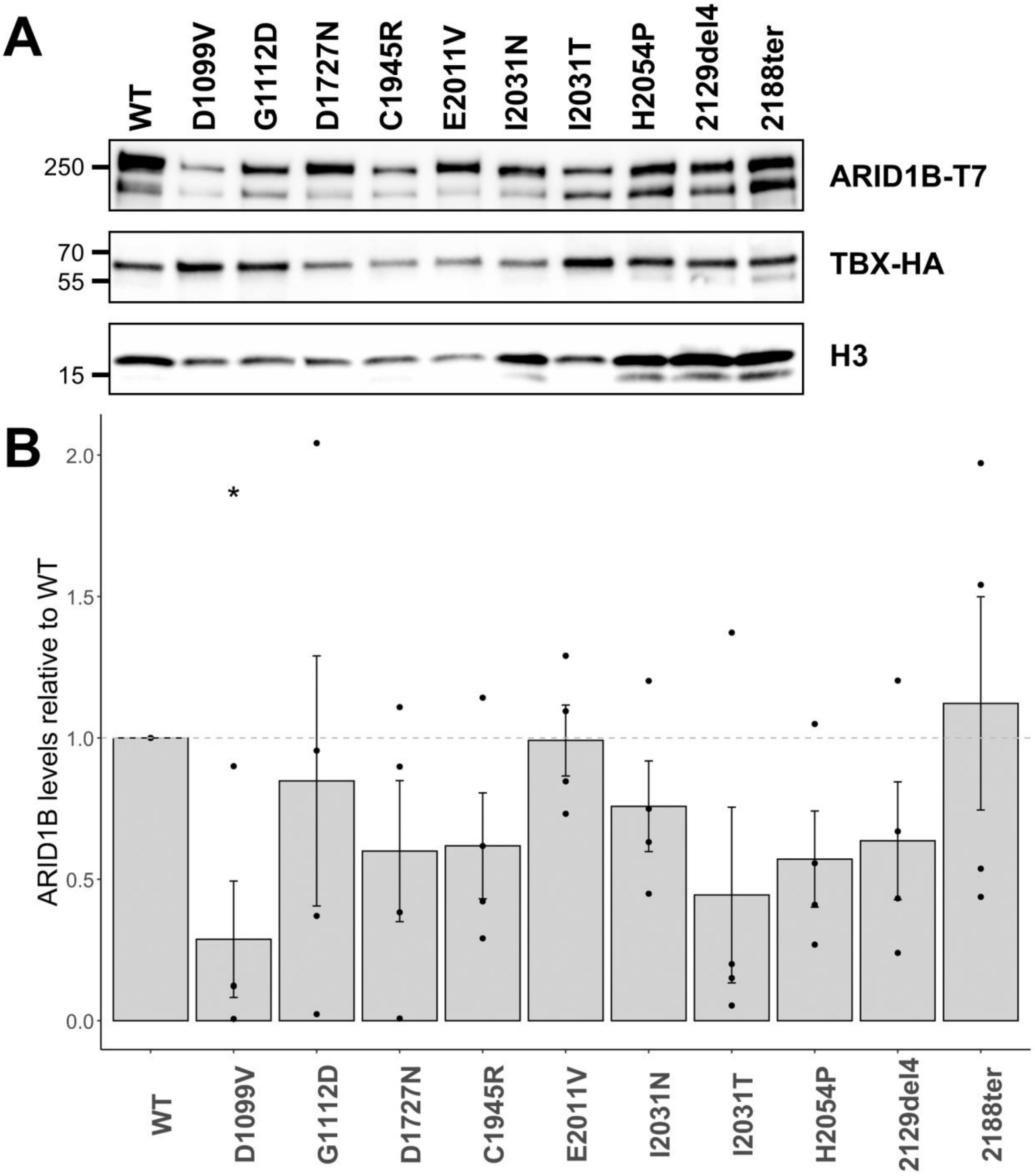

Fig. S4 | ARID1B protein aggregates are stable

ARID1B protein expression. **(A)** Representative western blot image. TBX1-HA was used as transfection control, Histone H3 as loading control. **(B)** Quantification of ARID1B protein levels. Wild type and mutant ARID1B-T7 was normalized to TBX1-HA and the value of wild type ARID1B-T7 was set to 1 (marked by dashed line). Data stems from 4 independent experiments. Sample means are depicted as bars with SEM, individual values as dots. P-values were calculated using a one sample t-test (hypothetical mean = 1, significance threshold < 0.05). * p < 0.05. Significant protein loss was only observed for variant D1099V, localized in the ARID domain.

SUPPLEMENTARY TABLES

Table S1 | Overview of ARID1B variants included in the study

	ID	cDNA (NM_020732.3)	Protein (NP_065783.3)	domain	CSS score methylation	CSS prediction	Variation ID (ClinVar)	allele frequency in gnomAD	dbSNP ID	Clinical significance (ClinVar)	Clinical significance (publication)	MetaRNN	source
1	D1099V	c.3296A>T	p.(Asp1099Val)	ARID	na	na	545058	NA	rs1554231259	likely pathogenic		moderate pathogenic	ClinVar
2	G1112D	c.3335G>A	p.(Gly1112Asp)	ARID	na	na	218776	NA	rs864309615	likely pathogenic		strong pathogenic	ClinVar
3	D1727N	c.5179G>A	p.(Asp1727Asn)	no domain	<0.01	no	2896505	0.000006815	rs771781497	VUS		strong benign	(Aref-Eshghi et al. 2018)
4	C1945R	c.5833T>C	p.(Cys1945Arg)	EHD2	0.88 (CSS1)	yes		NA	na	na		strong pathogenic	(Aref-Eshghi et al. 2018)
5	E2011V	c.6032A>T	p.(Glu2011Val)	EHD2	<0.01	no		0.000006575	rs1238364765	na		Uncertain	(Aref-Eshghi et al. 2018)
6	I2031N	c.6092T>A	p.(Ile2031Asn)	EHD2	na	na		NA	na	na	likely pathogenic	moderate pathogenic	(Yan et al. 2019)
7	I2031T	c.6092T>C	p.(Ile2031Thr)	EHD2	na	na	1254408	NA	rs2128397101	likely pathogenic		moderate pathogenic	(Mignot et al. 2016)/ClinVar
8	H2054P	c.6161A>C	p.(His2054Pro)	EHD2	na	na		NA	na	na	likely pathogenic	moderate pathogenic	(Miyamoto et al. 2021)
9	2129del4	c.6385_6397 delinsA	p.(Glu2129_Ala2133 delinsThr)	EHD2	CSS1	yes		NA	na	na		na	in house
10	2188ter	c.6463_6473del	p.(Ser2155Leufs*33)	EHD2	CSS1	yes		NA	na	na		na	(Hoyer et al. 2012; Vasileiou et al. 2015)

Table S2 | Waltz prediction for amylogenic regions in ARID1B

Positions	Sequence	Average score per residue
229-235	EFNNYYG	97.99
666-671	DLNLIQ	92.98
1100-1105	LFRLYV	96.32
1146-1152	IQYLFAF	97.04
1659-1664	NILLYD	98.33
1682-1688	LLVEYFR	97.99
1970-1980	GLVLILGKLIL	98.66
2027-2033	TLANISG	94.31
2114-2118	TLVRY	92.31
2240-2249	CDVLFQIGQL	92.98

Table S3 | *In silico* prediction scores for ARID1B variants

	ID	AM_Score	AM_prediction	VI_Score	VI_prediction	manual inspection	aggregation
1	D1099V	0.999	pathogenic	0.805	deleterious	na	***
2	G1112D	0.999	pathogenic	0.680	deleterious	na	***
3	D1727N	0.106	benign	0.393	neutral	na	ns
4	C1945R	0.998	pathogenic	0.632	deleterious	na	***
5	E2011V	0.556	ambiguous	0.523	deleterious	na	ns
6	I2031N	0.980	pathogenic	0.501	deleterious	na	***
7	I2031T	0.939	pathogenic	0.380	neutral	na	***
8	H2054P	0.995	pathogenic	0.830	deleterious	na	***
9	2129del4	na	na	na	na	deleterious	***
10	2188ter	na	na	na	na	deleterious	***

AM: AlphaMissense, VI: Vipur

Table S4 | Oligonucleotides for In-Fusion mutagenesis of pCMV-ARID1B-T7

Variant	Orient.	Sequence
**D1099V** c.3296A>T p.(Asp1099Val)	Fw	GCCCCTGGTCCTGTTCCGACTCTACGTCTGC
	Rev	AACAGGACCAGGGGCTTCTTGCCCACG
**G1112D** c.3335G>A p.(Gly1112Asp)	Fw	GATCGGGGATTTGGCCCAGGTTAATAAAAACAAG
	Rev	GCCAAATCCCCGATCTCTTTGACGCAG
**D1727N** c.5179G>A p.(Asp1727Asn)	Fw	TGGCAGACAATTCTGGGAAAGAGGAGGAAGATGC
	Rev	CAGAATTGTCTGCCAAGGACTGGCTG
**C1945R** c.5833T>C p.(Cys1945Arg)	Fw	CTAAGCGACGCATCTGTGTGTCCAATATTGTCCG
	Rev	AGATGCGTCGCTTAGCCAGCGAGTCC
**E2011V** c.6032A>T p.(Glu2011Val)	Fw	CAAAGATGTGTGGTGGTGGGACTGCCTC
	Rev	CACCACACATCTTTGCTGCAGGCCACC
**I2031N** c.6092T>A p.(Ile2031Asn)	Fw	GGCCAACAATTCCGGGCAGCTAGACTTGT
	Rev	CCGGAATTGTTGGCCAACGTGACCA
**I2031T** c.6092T>C p.(Ile2031Thr)	Fw	GGCCAACACTTCCGGGCAGCTAGACTTGT
	Rev	CCGGAAGTGTTGGCCAACGTGACCA
**H2054P** c.6161A>C p.(His2054Pro)	Fw	CTTGCTGCCCTGGATGGTGTGCCCGTC
	Rev	ATCCAGGGCAGCAAGCCATCCAAAATTGG
**2129del4** c.6386_6397del p.(Glu2129_Met2132del)	Fw	TCTGTCGAACGCTTTTATCGAACCTTGCCC
	Rev	AAAGCGTTCGACAGACTGGGTTTTTGCG
**2188ter** c.6463_6473del p.(Ser2155Leufs*33)	Fw	AGAAAGGACTTGATAAGCTTCCTAGAGGATGGG
	Rev	TATCAAGTCCTTTCTGCACAGCTATGGC

Table S5 | Antibodies and dilutions used

Antibody	Supplier & cat. no.	Method	Dilution
mouse monoclonal FLAG M2	Sigma F1804	IF/PLA	1:500
		Western	1:10,000
rabbit monoclonal T7	Cell Signalling Technologies #13246	IF/PLA	1:200
		Western	1:1,000/1:10,000
rat monoclonal Vimentin	Novus Biologicals 280618	IF	1:100
mouse monoclonal ɣ-Tubulin	Sigma T6557	IF	1:5,000
rabbit polyclonal HA	Sigma H6908	Western	1:100,000
rabbit monoclonal H3	Cell Signalling Technologies #4499	Western	1:1,000
goat anti rabbit Alexa Fluor 488	Molecular Probes A11008	IF	1:700
goat anti rabbit HRP	dianova 111-035-003 W	Western	1:10,000
goat anti mouse HRP	Invitrogen G-21040	Western	1:10,000

SUPPLEMENTARY REFERENCES

Aref-Eshghi E, Bend EG, Hood RL, et al (2018) BAFopathies' DNA methylation epi-signatures demonstrate diagnostic utility and functional continuum of Coffin–Siris and Nicolaides–Baraitser syndromes. Nat Commun 9:4885. https://doi.org/10.1038/s41467-018-07193-y

Hoyer J, Ekici AB, Endele S, et al (2012) Haploinsufficiency of ARID1B, a member of the SWI/SNF-a chromatin-remodeling complex, is a frequent cause of intellectual disability. Am J Hum Genet 90:565–572. https://doi.org/10.1016/j.ajhg.2012.02.007

Inoue H, Furukawa T, Giannakopoulos S, et al (2002) Largest Subunits of the Human SWI/SNF Chromatin-remodeling Complex Promote Transcriptional Activation by Steroid Hormone Receptors. Journal of Biological Chemistry 277:41674–41685. https://doi.org/10.1074/jbc.M205961200

Mignot C, Moutard M-L, Rastetter A, et al (2016) *ARID1B* mutations are the major genetic cause of corpus callosum anomalies in patients with intellectual disability. Brain 139:e64–e64. https://doi.org/10.1093/brain/aww181

Miyamoto S, Kato M, Hiraide T, et al (2021) Comprehensive genetic analysis confers high diagnostic yield in 16 Japanese patients with corpus callosum anomalies. J Hum Genet 66:1061–1068. https://doi.org/10.1038/s10038-021-00932-y

Vasileiou G, Ekici AB, Uebe S, et al (2015) Chromatin-Remodeling-Factor ARID1B Represses Wnt/β-Catenin Signaling. The American Journal of Human Genetics 97:445–456. https://doi.org/10.1016/j.ajhg.2015.08.002

Wittmann M-T, Katada S, Sock E, et al (2021) scRNA sequencing uncovers a TCF4-dependent transcription factor network regulating commissure development in mouse. Development 148:dev196022. https://doi.org/10.1242/dev.196022

Xi Q, He W, Zhang XH-F, et al (2008) Genome-wide Impact of the BRG1 SWI/SNF Chromatin Remodeler on the Transforming Growth Factor β Transcriptional Program. Journal of Biological Chemistry 283:1146–1155. https://doi.org/10.1074/jbc.M707479200

Yan H, Shi Z, Wu Y, et al (2019) Targeted next generation sequencing in 112 Chinese patients with intellectual disability/developmental delay: novel mutations and candidate gene. BMC Med Genet 20:80. https://doi.org/10.1186/s12881-019-0794-y
